# Association Between Adherence Barriers, Self‐Reported Adherence, and Psychiatric Symptoms Among Adults With Bipolar Disorder

**DOI:** 10.1111/bdi.70067

**Published:** 2025-11-11

**Authors:** Martha Sajatovic, Jennifer B. Levin, Clara Adeniyi, Jessica Black, Celeste Weise, Nicole Fiorelli, Erika Kelley, Doug Einstadter, Mark Bauer, Farren B. S. Briggs

**Affiliations:** ^1^ Department of Psychiatry, Case Western Reserve University School of Medicine University Hospitals Cleveland Medical Center Cleveland Ohio USA; ^2^ Neurological and Behavioral Outcomes Center University Hospitals Cleveland Medical Center Cleveland Ohio USA; ^3^ Department of Obstetrics and Gynecology University Hospitals Cleveland Medical Center Cleveland Ohio USA; ^4^ Department of Reproductive Biology Case Western Reserve University Cleveland Ohio USA; ^5^ Center for Health Care Research and Policy MetroHealth System Cleveland Cleveland Ohio USA; ^6^ Department of Medicine and Population and Quantitative Health Sciences Case Western Reserve University School of Medicine Cleveland Ohio USA; ^7^ Department of Psychiatry Harvard Medical School Boston Massachusetts USA; ^8^ Department of Public Health Sciences University of Miami Miller School of Medicine Miami Florida USA

**Keywords:** adherence barriers, bipolar disorder, manic depressive disorder, medication adherence

## Abstract

**Aims:**

While suboptimal medication adherence is a widely recognized problem for people with bipolar disorder (BD), it is less clear how individual characteristics are associated with varying levels of adherence and how specific adherence barriers impact behaviors. This interim analysis from an ongoing randomized controlled trial (RCT) examined associations between patient‐reported adherence, adherence barriers, and mood symptoms among poorly adherent individuals with BD.

**Methods:**

RCT participants were adults age ≥ 18 years old with BD Type 1 or 2, difficulties with medication adherence, and actively symptomatic as measured by Brief Psychiatric Rating Scale (BPRS) score ≥ 36, Young Mania Rating Scale (YMRS) > 8, or Montgomery Asberg Depression Rating Scale (MADRS) > 8. Adherence was assessed using the self‐reported Tablets Routine Questionnaire (TRQ) and grouped into 3 clinically relevant groups: those with TRQs < 20% (good adherence), ≥ 20% and < 50% (fair adherence), and ≥ 50% (poor adherence). Adherence barriers were assessed with the Oxford Bipolar Knowledge Questionnaire (OBQ), Self‐Report Habit Index (SRHI), Communication Styles Scale (CSS), and Stages of Change Readiness and Treatment Eagerness Scale (SOCRATES 8A).

**Results:**

Analysis was derived from screening and baseline data on the first 129 randomized participants. Mean age was 42.18 (SD = 13.04) years, with 76.74% (*n* = 99) female and 41.09% (*n* = 53) non‐White. The mean past 7‐day percentage of days with missed BD medications using TRQ was 34.34% (SD = 30.32) at screening and 24.82% (SD = 27.70) at baseline. The average time between screening visit and baseline was 18.90 (SD = 12.46) days. Comparing adherence groups, MADRS and BPRS were significantly higher in those with worse adherence both at screening and baseline (*p* < 0.05 for all). With respect to BD adherence barriers, only SRHI was significantly inversely correlated with TRQ at screening (*p* < 0.001) and both SRHI and SOCRATES 8A (Taking steps sub‐scale) were significantly inversely correlated with TRQ at baseline (*p* = 0.001 and *p* = 0.022, respectively).

**Conclusions:**

Poorly adherent individuals with BD have significantly more severe global psychopathology and worse depressive severity. Significant adherence barriers include lifestyle routines that do not promote regular medication‐taking and engagement in reducing the use of substances. Given the extensive burden of poor adherence in BD, adherence promotion efforts should target specific and actionable barriers.

**Trial Registration:**

ClinicalTrials.gov identifier: NCT04622150

## 1 Introduction

Sub‐optimal adherence with BD medications is common among people with bipolar disorder (BD) and is associated with multiple poor outcomes, including reduced quality of life, high rates of relapse, and premature mortality, including suicide [1]. A 2018 narrative review of adherence factors among people with BD noted that drivers of poor adherence include adverse effects of medication, complex medication regimens, negative patient attitudes towards medication, poor insight, rapid‐cycling mood states, comorbid substance use problems, and a poor therapeutic alliance [1]. This same review suggested that adherence promotion likely requires a collaborative relationship between people with BD and their healthcare providers, such that implemented strategies need to be patient specific, reflecting the fact that nonadherence has no single cause and cannot be addressed with a one‐size‐fits‐all approach [1].

Medication adherence levels in people with BD can vary widely. A 2023 nation‐wide Finnish cohort of over 33,000 individuals with BD found that 59.1% had at least one non‐dispensed mood stabilizer or antipsychotic prescription and 31.0% were non‐adherent to ≥ 20% of their mood stabilizer and/or antipsychotic prescriptions [2]. There is also variation among individuals who are considered sub‐optimally adherent (typically defined as missing > 20% of prescribed medication) [2] and samples with differing adherence cut‐points may have different demographic and clinical features [3, 4].

Customized Adherence Enhancement (CAE) is a novel behavioral approach intended to improve medication adherence in people with serious mental illness, including bipolar disorder [5, 6, 7]. The premise of CAE is that therapeutic focus is targeted at those adherence barriers which are identified by a given individual with BD. The CAE approach, based on an extensive body of mixed‐methods research [6, 8, 9, 10], targets 4 of the most common barriers to medication adherence among those with BD: (1) a limited or inaccurate knowledge of the role of medications in helping people with BD to manage their health, (2) unstable lifestyle routines that lead to missed medication‐taking, (3) poor communication with care providers, especially in relation to medication prescribing and side effect management, and (4) substance abuse as an impediment to regular engagement in care/medication‐taking [6]. A recent international effort by McIntyre and colleagues [11] has called for the need to identify clinically meaningful subgroups of persons with BD who may respond differentially to specific treatments at the point of care. They also suggested that characterizing select domains in BD provides actionable information and guides shared decision making.

In spite of a body of literature that demonstrates the efficacy of CAE in improving medication adherence and psychiatric symptoms, there have been no analyses that specifically evaluate adherence barriers assessed via standardized scales. Aligning with the call to action for personalization of care in BD [11], a better understanding of adherence barriers among people with BD who have suboptimal adherence is also relevant and generalizable to psychotherapeutic approaches that target self‐management and patient empowerment. This interim analysis from an ongoing randomized controlled trial (RCT) examined associations between patient‐reported medication adherence, adherence barriers measured using standardized rating scales and mood symptoms among poorly adherent individuals with BD.

## 2 Methods

### 2.1 Overall Study

This analysis stems from an ongoing US National Institute of Mental Health (NIMH)‐funded RCT to evaluate an intervention aimed at enhancing medication adherence in symptomatic individuals with BD. The overall intent of the ongoing RCT is to compare adherence and clinical outcomes associated with CAE versus Enhanced Treatment as Usual (ETAU) in individuals with BD who acknowledge adherence challenges. CAE is a brief and practical psychotherapeutic intervention that identifies the unique adherence barriers of individual patients and then addresses these areas for intervention through a flexible modular format. A detailed description of the overall project has been described elsewhere [12]. The current analysis was derived from the study's screening and baseline data for the first 129 randomized participants.

## 3 Sample

The RCT inclusion criteria included having a diagnosis of BD type 1 or type 2 for at least 2 years and being prescribed at least one evidence‐based mood stabilizer (lithium, anticonvulsants, antipsychotics) for at least 6 months. Participant BD diagnosis was confirmed using the Structured Clinical Interview for DSM‐5 patient version (SCID‐5‐RV) [13]. All participants had current or past difficulties with medication adherence or had indicated that they sometimes tried to manage their BD on their own without medication. The study was approved by the local institutional review board (IRB) and all study participants provided written, informed consent. Study data were collected and managed using Research Electronic Data Capture (REDCap), an electronic data capture tool hosted at Case Western Reserve University [14].

## 4 Measures

Demographic and clinical information collected included age, sex, race/ethnicity, marital status, living situation, years of education, socioeconomic status, and self‐reported family and personal psychiatric history. Substance use over the past year was assessed using the Alcohol Use Disorders Identification Test—Self‐Report Version (AUDIT) [15] and the Drug Abuse Screening Test (DAST‐10) [16]. The AUDIT is a 10‐item measure used to screen for excessive alcohol use, with higher scores reflecting more harmful alcohol use [17]. The DAST‐10 is an abbreviated version of the original 28‐item DAST and measures drug‐related problems [16]. Higher scores reflect more severe problems related to drug abuse.

### 4.1 Medication Adherence and Adherence Barriers

Self‐reported medication adherence was assessed using the Tablets Routine Questionnaire (TRQ) [18], which assessed the percent of missed doses for each medication over the past 7 and 30 days. If a participant was on more than one medication to treat BD, an average TRQ score was calculated. Scores range from 0 (missing no medications) to 100 (missing all medications), with higher TRQ scores indicating worse adherence. For this analysis, optimizing participant recall accuracy, we focused on the past 7‐day TRQ. Self‐reported medication adherence was assessed at study screening when individuals were assessed for eligibility and again at the baseline visit prior to being randomized into the RCT. Adherence was categorized into 3 clinically relevant groups: those with TRQs < 20% (good adherence), ≥ 20% and < 50% (fair adherence), and ≥ 50% (poor adherence). The average time between the screening visit and baseline was 18.9 (SD = 12.5) days.

As the CAE intervention features flexibly delivered modules focusing on the most common and potentially modifiable barriers to medication adherence among people with BD, RCT participants were assessed on 4 key patient‐level barriers at baseline. The barrier of inadequate knowledge of BD was assessed with the Oxford Bipolar Knowledge Questionnaire (OBQ), a 40‐item self‐report tool. The OBQ uses a 3‐point Likert scale to assess BD knowledge domains [19]. The barrier of lack of routines as an impediment to regular medication‐taking was assessed using the Self‐Report Habit Index (SRHI), a 12‐item self‐report measure of habit strength [20]. The SRHI is often used in measuring unhealthy habits such as smoking, but in our study was used to measure the strength of medication‐taking. Higher scores are consistent with high automaticity and a strong integration of medication taking into one's life, while lower scores represent challenges with consistency. The SRHI assesses 3 characteristics of habitual action that include automaticity (how much an individual has to think about before initiating an action), frequency (how often something occurs), and relevance (how much an individual feels a characteristic is typical for them) [21, 22]. The barrier of inadequate communication with providers was assessed using the Communication Styles Scale (CSS), a 9‐item patient‐rated measure of the impact of prescribing clinician communication style on medication beliefs and adherence behavior in depressed patients [23]. The barrier of ongoing substance abuse and no/minimal motivation to change was assessed using the 19‐item Stages of Change Readiness and Treatment Eagerness Scale (SOCRATES 8A) [24]. Higher scores indicate stronger agreement with stage of change.

In addition to the 4 barriers that are targeted by the CAE intervention, we examined the Attitude Towards Mood Stabilizers Questionnaire (AMSQ), a 19‐item measure evaluating an individual's attitude towards mood stabilizers. Scores range from 0 to 19, with higher scores reflecting more negative attitudes [25]. We also examined the non‐adherence subscale of the Rating of Medication Influences (ROMI), a scale designed to measure an individual's reasons for medication compliance and noncompliance [26]. Scoring on this subscale of the ROMI ranges from 0 to 30, with higher scores indicating more reasons for non‐adherence.

### 4.2 Psychiatric Symptoms

Baseline mood and other psychiatric symptoms were assessed with the Young Mania Rating Scale (YMRS) [27], the Montgomery Asberg Depression Rating Scale (MADRS) [28], and the Brief Psychiatric Rating Scale (BPRS) [29]. The YMRS is an 11‐item scale that is used to measure the severity of hypomania/mania in patients with bipolar disorder [27]. Higher scores indicate more severe manic symptoms. The MADRS is a 10‐item rating scale of depressive symptoms [30]. Higher scores indicate more severe depressive symptoms. The BPRS is a 24‐item rating scale that assesses a broad range of symptoms of serious mental illness [29, 31]. Higher scores indicate more severe symptomatology.

## 5 Data Analysis

The objective of the analysis was to examine clinically relevant sub‐samples with differing levels of self‐reported medication adherence (individuals with good, fair, and poor adherence) at RCT screening and baseline timepoints, and to identify associations between adherence, adherence barriers, and BD symptoms. First, descriptive statistics summarized adherence (TRQ at screening and at baseline) and clinical and demographic attributes of participants who completed baseline evaluations. Comparisons across adherence subgroups were conducted using Fisher's exact test for categorical variables and Kruskal–Wallis tests for continuous variables. Non‐parametric Spearman rank correlations explored relationships among adherence scores and adherence barrier scores. A two‐sided alpha of 0.05 was considered statistically significant.

## 6 Results

### 6.1 Overall Sample Description

The analysis sample was derived from screening and baseline data on the first 129 randomized participants. Table 1a,b shows the demographic, adherence, and psychiatric symptom data at screening and baseline timepoints. Mean age was 42.2 (SD = 13.0) years, with 76.74% (*n* = 99) female and 41.1% (*n* = 53) non‐White. Mean past 7‐day percentage of days with missed BD medications using TRQ was 34.3% (SD = 30.3) and 24.8% (SD = 27.7) at screening and baseline, respectively. The average time between screening visit and baseline was 18.9 (SD = 12.5) days. Several points are worth noting regarding the sample screening and baseline characteristics, including the fact that mean levels of BD manic symptoms were relatively low, with a mean of 8.9 (SD = 5.2); mean levels of BD depressive symptoms were moderate, and only a minority (20.2%) were men.

**TABLE 1:** Characteristics of trial participants with differing levels of medication adherence (a) at screening and (b) at baseline.

Variable	*N*	Total sample	*N*	TRQ < 20% (good adherence)	*N*	TRQ ≥ 20% < 50% (fair adherence)	*N*	TRQ ≥ 50% (poor adherence)	*p*
**(a)**									
Age, mean, SD, range	129	42.18 (13.04), 18–71	50	42.78 (13.81), 20–71	45	43.62 (11.16), 18–64	34	39.38 (14.09), 21–70	0.23
Gender									
Female		99 (76.74%)		38 (76.00%)		37 (82.22%)		24 (70.59%)	0.71
Male		26 (20.16%)		11 (22.00%)		7 (15.56%)		8 (23.53%)	
Other		4 (3.10%)		1 (2.00%)		1 (2.22%)		2 (5.88%)	
Marital status									
Married or cohabitating		43 (33.33%)		16 (32.00%)		18 (40.00%)		9 (26.47%)	0.85
Single, never married		58 (44.96%)		24 (48.00%)		19 (42.22%)		15 (44.12%)	
Divorced		22 (17.05%)		8 (16.00%)		6 (13.33%)		8 (23.53%)	
Separated/widowed		6 (4.65%)		2 (4.00%)		2 (4.44%)		2 (5.88%)	
Race									
Black		29 (22.48%)		8 (16.00%)		12 (26.67%)		9 (26.47%)	0.69
White		76 (58.91%)		31 (62.00%)		26 (57.78%)		19 (55.88%)	
Other		24 (18.60%)		11 (22.00%)		7 (15.56%)		6 (17.65%)	
Ethnicity									
Hispanic/Latino		20 (15.50%)		6 (12.00%)		10 (22.22%)		4 (11.76%)	0.27
Living situation									
Alone		32 (24.81%)		13 (26.00%)		10 (22.22%)		9 (26.47%)	0.90
Significant other		43 (33.33%)		17 (34.00%)		15 (33.33%)		11 (32.35%)	1.00
Family		63 (48.84%)		20 (40.00%)		21 (46.67%)		22 (64.71%)	0.09
Other (e.g., roommate)		11 (8.53%)		6 (12.00%)		4 (8.89%)		1 (2.94%)	0.38
Education (in years)	129	13.54 (2.37), 8–20	50	14.10 (2.43), 10–20	45	13.00 (2.30), 8–19	34	13.44 (2.25), 10–20	0.058
Occupation									
Full time		31 (24.03%)		15 (30.00%)		8 (17.78%)		8 (23.53%)	0.58
Part time		32 (24.81%)		14 (28.00%)		12 (26.67%)		6 (17.65%)	
Unemployed (expected to work)		15 (11.63%)		4 (8.00%)		5 (11.11%)		6 (17.65%)	
Unemployed (disabled)		51 (39.53%)		17 (34.00%)		20 (44.44%)		14 (41.18%)	
Bipolar diagnosis									
Type 1		102 (79.07%)		42 (84.00%)		34 (75.56%)		26 (76.47%)	0.54
Type 2		27 (20.93%)		8 (16.00%)		11 (24.44%)		8 (23.53%)	
Age of bipolar disorder onset	129	21.49 (11.48), 1–65	50	20.38 (9.08), 4–50	45	22.40 (12.22), 1–50	34	21.91 (13.65), 5–65	0.86
Positive family history for mental illness		109 (84.50%)		46 (92.00%)		36 (80.00%)		27 (79.41%)	0.18
Number of previous psychiatric hospitalizations	129	3.31 (3.94), 0–20	50	3.70 (4.09), 0–15	45	2.91 (3.50), 0–15	34	3.26 (4.32), 0–20	0.59
Comorbid substance abuse									
AUDIT	116	4.09 (7.51), 0–34	48	4.40 (7.96), 0–30	36	4.22 (7.81), 0–34	32	3.47 (6.62), 0–32	0.68
DAST‐10	119	1.90 (2.77), 0–10	47	2.06 (2.81), 0–10	39	2.23 (3.09), 0–10	33	1.27 (2.25), 0–8	0.10
Bipolar disorder symptoms at screening									
YMRS	127	8.86 (5.18), 1–29	48	8.90 (6.00), 1–29	45	9.20 (4.74), 1–19	34	8.35 (4.57), 1–19	0.64
MADRS	129	23.72 (9.36), 4–43	50	22.14 (8.59), 8–42	45	22.58 (9.66), 4–42	34	27.56 (9.23), 11–43	**0.031** *
BPRS	128	37.19 (7.41), 20–60	49	36.57 (8.18), 23–60	45	35.84 (6.60), 20–51	34	39.85 (6.75), 25–54	**0.040** *
**(b)**									
Age mean, SD, range	129	42.18 (13.04), 18–71	74	43.01 (13.20), 20–70	35	41.51 (12.71), 18–71	20	40.25 (13.39), 21–66	0.67
Gender									
Female		99 (76.74%)		58 (78.38%)		24 (68.57%)		17 (85.00%)	0.33
Male		26 (20.16%)		15 (20.27%)		8 (22.86%)		3 (15.00%)	
Other		4 (3.10%)		1 (1.35%)		3 (8.57%)		0 (0.00%)	
Marital status									
Married or cohabitating		43 (33.33%)		28 (37.84%)		10 (28.57%)		5 (25.00%)	0.39
Single, never married		58 (44.96%)		33 (44.59%)		17 (48.57%)		8 (40.00%)	
Divorced		22 (17.05%)		11 (14.86%)		7 (20.00%)		4 (20.00%)	
Separated/widowed		6 (4.65%)		2 (2.70%)		1 (2.86%)		3 (15.00%)	
Race									
Black		29 (22.48%)		15 (20.27%)		8 (22.86%)		6 (30.00%)	0.40
White		76 (58.91%)		46 (62.16%)		22 (62.86%)		8 (40.00%)	
Other		24 (18.60%)		13 (17.57%)		5 (14.29%)		6 (30.00%)	
Ethnicity									
Hispanic/Latino		20 (15.50%)		9 (12.16%)		8 (22.86%)		3 (15.00%)	0.15
Living situation									
Alone		32 (24.81%)		16 (21.62%)		9 (25.71%)		7 (35.00%)	0.42
Significant other		43 (33.33%)		29 (39.19%)		10 (28.57%)		4 (20.00%)	0.24
Family		63 (48.84%)		36 (48.65%)		16 (45.71%)		11 (55.00%)	0.83
Other (e.g., roommate)		11 (8.53%)		7 (9.46%)		4 (11.43%)		0 (0.00%)	0.34
Education (in years)	129	13.54 (2.37), 8–20	74	13.53 (2.42), 8–20	35	13.40 (2.08), 10–19	20	13.85 (2.70), 10–20	0.88
Occupation									
Full time		31 (24.03%)		21 (28.38%)		6 (17.14%)		4 (20.00%)	0.27
Part time		32 (24.81%)		19 (25.68%)		11 (31.43%)		2 (10.00%)	
Unemployed (expected to work)		15 (11.63%)		6 (8.11%)		4 (11.43%)		5 (25.00%)	
Unemployed (disabled)		51 (39.53%)		28 (37.84%)		14 (40.00%)		9 (45.00%)	
Bipolar diagnosis									
Type 1		102 (79.07%)		60 (81.08%)		26 (74.29%)		16 (80.00%)	0.69
Type 2		27 (20.93%)		14 (18.92%)		9 (25.71%)		4 (20.00%)	
Age of bipolar disorder onset	129	21.49 (11.48), 1–65	74	21.84 (12.13), 4–65	35	20.20 (7.73), 6–36	20	22.45 (14.52), 1–50	1.00
Positive family history for mental illness		109 (84.50%)		64 (86.49%)		27 (77.14%)		18 (90.00%)	0.37
Number of previous psychiatric hospitalizations	129	3.31 (3.94), 0–20	74	2.89 (3.57), 0–15	35	3.71 (3.58), 0–12	20	4.15 (5.58), 0–20	0.29
Comorbid substance abuse									
AUDIT	116	4.09 (7.51), 0–34	69	4.29 (7.83), 0–34	31	4.06 (8.24), 0–32	16	3.25 (4.23), 0–14	0.36
DAST‐10	119	1.90 (2.77), 0–10	68	2.26 (3.13), 0–10	33	1.61 (2.40), 0–9	18	1.06 (1.59), 0–6	0.38
Bipolar disorder symptoms at baseline									
YMRS	129	8.50 (5.77), 1–28	74	7.99 (5.93), 1–28	35	8.51 (4.73), 2–22	20	10.35 (6.67), 1–25	0.20
MADRS	129	20.43 (9.78), 1–41	74	17.86 (8.74), 1–36	35	23.20 (10.21), 6–41	20	25.05 (10.10), 5–40	**0.004** *
BPRS	129	34.35 (7.97), 20–57	74	32.72 (7.11), 20–50	35	35.29 (8.50), 20–57	20	38.75 (8.46), 25–57	**0.014** *

*Note:* To determine the *p* values, the Kruskal–Wallis test was used for continuous variables, and Fisher's exact test was used for categorical variables. For the “Living situation” categories, participants were able to select more than one option, so the options were analyzed individually.

Abbreviations: AUDIT, Alcohol Use Disorders Identification Test—Self‐Report Version; BPRS, Brief Psychiatric Rating Scale; DAST, Drug Abuse Screening Test; MADRS, Montgomery Asberg Rating Scale; TRQ, Tablets Routine Questionnaire; YMRS, Young Mania Rating Scale.

* Significance at the 0.05 level.

### 6.2 Adherence Sub‐Groups

Comparing adherence groups at the screening timepoint (Table 1a), the sample had 38.8% with good adherence (*N* = 50), 34.9% (*N* = 45) with fair adherence, and 26.3% (*N* = 34) with poor adherence. There were more similarities than differences across the adherence sub‐groups, including no differences in demographic or clinical characteristics except for BD symptom severity. The distribution of screening total scores on MADRS and BPRS significantly differed across adherence subgroups, with higher means in those with worse adherence at screen (*p* = 0.031 for MADRS, *p* = 0.040 for BPRS). The distribution of YMRS scores was not different across subgroups.

Comparing adherence groups at the baseline timepoint (Table 1b), the sample had 57.4% with good adherence (*N* = 74), 27.1% (*N* = 35) with fair adherence, and 15.5% (*N* = 20) with poor adherence. Similar to the screening sample, there were few differences in demographic or clinical characteristics. The distribution of mean total scores on MADRS and BPRS at baseline was significantly higher in those with worse adherence (*p* = 0.004 for MADRS, *p* = 0.014 for BPRS). As with the screening sample, the distribution of YMRS scores was not different across the adherence subgroups.

### 6.3 Correlations of Adherence Barriers and Self‐Reported Adherence

Table 2 illustrates correlation analyses between TRQ and the 4 key adherence barriers targeted in our RCT intervention. With respect to BD adherence barriers, only SRHI was significantly negatively correlated with TRQ at screening (*p* < 0.001), and both SRHI and SOCRATES 8A (Taking steps sub‐scale) were significantly negatively correlated with TRQ at baseline (*p* = 0.001 and *p* = 0.022, respectively), such that less automaticity and less motivation for change were associated with worse adherence. There was no association between TRQ and CSS or OBQ. Both the AMSQ and the ROMI items relating to reasons for worse adherence were significantly correlated in the expected direction of worse attitudes towards medication and more reasons for non‐adherence (*p* = 0.013) in those with poorer adherence at both screening and baseline (*p* < 0.001 at both screening and baseline AMSQ, *p* = 0.013 screening ROMI and *p* = 0.016 baseline ROMI).

**TABLE 2:** Spearman Rank Correlations between adherence barriers and adherence among patients with bipolar disorder.

Variables	TRQ screen		TRQ baseline	
	*r*	*p*	*r*	*p*
TRQ screen	—	—	—	—
TRQ baseline	0.58	**< 0.001** *	—	—
OBQ	−0.03	0.76	−0.07	0.52
SRHI	−0.38	**< 0.001** *	−0.30	**< 0.001** *
CSS	−0.16	0.086	−0.12	0.19
SOCRATES 8 (recognition)	−0.10	0.40	−0.21	0.077
SOCRATES 8 (Ambivalence)	−0.06	0.64	−0.23	0.058
SOCRATES 8 (taking steps)	−0.10	0.42	−0.28	**0.022** *
AMSQ	0.49	**< 0.001** *	0.36	**< 0.001** *
ROMI	0.23	**0.013** *	0.23	**0.016** *

Abbreviations: AMSQ, Attitude towards Mood Stabilizers Questionnaire; CSS, Communications Styles Scale; OBQ, Oxford Bipolar Knowledge Questionnaire; ROMI, Rating of Medication influences, reasons for non‐adherence subscale; SOCRATES 8, Stages of Charge Readiness and Treatment Eagerness Scale; SRHI, Self‐report Habit Index; TRQ, Tablets Routine Questionnaire.

* Significance at the 0.05 level.

## 7 Discussion

This analysis of a screening and baseline sample of poorly adherent patients with BD participating in a clinical trial found that BD symptom severity was significantly associated with self‐reported medication treatment adherence, while demographic and other clinical features such as gender, race, ethnicity, and Type 1 versus 2 BD were not associated with adherence. Individuals with worse adherence had worse attitudes towards medication and more reasons for poor adherence. These findings have clinical relevance given the sample is generalizable to real‐world populations with adherence challenges. Study inclusion criteria required individuals to acknowledge adherence problems; the sample was drawn from individuals that use US public‐sector mental health care, and approximately 40% of the sample was non‐White. Consistent with other research reports [32], the sample reporting “good” adherence (missing < 20% of prescribed BD medication) increased from 38.8% to 57.4% in the approximately 2‐week time interval between screening and baseline, likely a Hawthorne effect of adherence monitoring. In line with our findings, nonadherence rates of approximately 50% are commonly reported for patients with BD [18, 33, 34] and in a multi‐country European survey of over 2000 psychiatrists estimated that the majority of patients (57%) with BD were nonadherent or partially adherent to treatment [35].

In contrast to our current findings of no demographic differences across groups with various levels of adherence, some previous literature has noted demographic risk factors for poor adherence, including gender, race, and level of education [34, 36]. Aligned with our findings, a literature review by Jawad and colleagues on adherence in BD [1] concluded that sociodemographic factors, including socioeconomic status, do not appear to be major determinants of adherence. An important caveat to conclusions from our report is the fact that men with BD were poorly represented in our sample (20% of the total).

The literature review on adherence in BD by Jawad and colleagues [1] noted the importance of “course of illness” on adherence, especially as it may fluctuate over time. Lower levels of adherence may be associated with greater illness complexity and complications such as psychiatric comorbidity, suicide attempts, and resistant BD symptoms [37, 38, 39]. Our current analysis found that more severe depression and global psychopathology were associated with worse medication adherence, but that manic severity was not associated with adherence levels. It is possible that we failed to find a significant association between manic symptoms and adherence because of the relatively low manic symptom severity in our sample. Other reports have used YMRS score total cut‐offs of < 10 or < 7 to categorize BD manic remission [40, 41]. Our mean baseline YMRS score was 8.5. In contrast, our total mean sample baseline levels of depression were relatively high with a mean of 20.4. For BD depression, remission has traditionally been defined as ≤ 12 on MADRS [42].

With respect to adherence barriers, our analysis found that habit routines were significantly associated with adherence levels such that individuals who self‐identified more habitual action also reported being more adherent with BD medications. A systematic literature review and meta‐analysis focused on the SRHI in relation to nutrition and physical activity behaviors by Gardner and colleagues [21] found that habit moderated the relationship between intention and behavior, such that intentions had reduced impact on behavior where habit was strong. Building medication‐taking into a habitual or automatic activity might be particularly important for individuals with BD given the expected mood and polarity fluctuations characteristic of bipolar illness.

In addition to habit formation, taking steps to minimize the use of drugs or alcohol was associated with better adherence to BD medications. Substance use comorbidity has been demonstrated to be a risk factor for poor adherence to BD medication treatment [43]. Other reports have noted the importance of actively addressing substance use and BD at the same time to optimize health outcomes [8]. This may require collaborative work between psychiatry generalists and addiction services to increase patient awareness of substance misuse as a potential BD relapse indicator and could help to promote longer‐term engagement and adherence to both BD and substance use disorder treatments.

Interestingly, our findings did not suggest that knowledge of BD and communication with prescribing providers were associated with adherence. Psychoeducation is an evidence‐based psychotherapy for BD that is premised upon the notion that improving knowledge about BD and BD medication can improve medication adherence along with multiple other health outcomes [44]. However, there is also an abundant literature in behavioral medicine noting that health knowledge alone may not be sufficient to drive health behavioral change [45]. There is also literature that supports the positive association of therapeutic alliance and good communication as a driver of engagement and adherence to BD treatment. A previous study of over 3000 patients with BD found that patients' perceptions of collaboration, empathy, and accessibility of their psychiatrist were significantly associated with adherence to treatment [46]. Given that patients may have multiple clinicians involved in their care, the question could be raised as to whether participants completed the CSS with their prescribing clinician in mind. To help clarify this in the conduct of our study, we specifically asked individuals to refer to “The clinician who prescribes your medication for bipolar disorder.”

While our analysis had some key findings with potentially important clinical implications, there are also limitations to our study that could limit broader application to promoting adherence among people with BD. A self‐reported adherence level could overestimate actual adherence behavior. Small sample size and a single geographic location could also impact generalizability. While the study inclusion required individuals to acknowledge medication adherence challenges, it is likely that those with the least likelihood of engaging in care for BD would not volunteer to participate in a research study. Additionally, because the CAE intervention focused on the main barriers of BD knowledge, medication routines, communication with providers, and substance use, findings may not generalize to other medication adherence barriers that may occur among people with BD.

In conclusion, poorly adherent individuals with BD have significantly more severe global psychopathology and worse depressive severity compared to those with better adherence. Significant medication adherence barriers include lifestyle routines that do not promote regular medication‐taking and issues related to substance use disorders. Given the extensive burden of suboptimal adherence on people with BD, their families, and on society, adherence promotion efforts should target specific and actionable barriers.

## Conflicts of Interest

Martha Sajatovic: Research grants within the past 3 years: Intra‐Cellular, Merck, Otsuka, Alkermes, International Society for Bipolar Disorders (ISBD), National Institutes of Health (NIH), Centers for Disease Control and Prevention (CDC), Patient‐Centered Outcomes Research Institute (PCORI). Consultant in the past year: Alkermes, Otsuka, Janssen, Lundbeck, Teva, Neurelis. Farren B.S. Briggs: Speaker fees from Sanofi. Jennifer B. Levin: Research grants within the past 3 years: Merck, National Institutes of Health (NIH), American Heart Association (AHA). Erika Kelley: Research grants within the past 3 years: The Patty Brisben Foundation. The other authors declare no conflicts of interest.

## Data Availability

This study data is entered into the common informatics platform by the U.S. National Institute of Mental Health (NIMH), National Database for Clinical Trials Related to Mental Illness (http://ndct.nimh.nih.gov, NDCT).
